# Copy Number Variation of KIR Genes Influences HIV-1 Control

**DOI:** 10.1371/journal.pbio.1001208

**Published:** 2011-11-29

**Authors:** Kimberly Pelak, Anna C. Need, Jacques Fellay, Kevin V. Shianna, Sheng Feng, Thomas J. Urban, Dongliang Ge, Andrea De Luca, Javier Martinez-Picado, Steven M. Wolinsky, Jeremy J. Martinson, Beth D. Jamieson, Jay H. Bream, Maureen P. Martin, Persephone Borrow, Norman L. Letvin, Andrew J. McMichael, Barton F. Haynes, Amalio Telenti, Mary Carrington, David B. Goldstein, Galit Alter, on behalf of NIAID Center for HIV/AIDS Vaccine Immunology (CHAVI)

**Affiliations:** 1 Center for Human Genome Variation, Duke University School of Medicine, Durham, North Carolina, United States of America; 2 Global Health Institute, School of Life Sciences, Ecole Polytechnique Fédérale de Lausanne, Lausanne, Switzerland; 3 Department of Biostatistics and Bioinformatics, Duke University, Durham, North Carolina, United States of America; 4 Institute of Clinical Infectious Diseases, Catholic University of the Sacred Heart, Rome, Italy; 5 Division of Infectious Diseases, Siena University Hospital, Siena, Italy; 6 irsiCaixa Foundation and Hospital Germans Trias i Pujol, Badalona, Spain; 7 Institució Catalana de Recerca i Estudis Avançats, Barcelona, Spain; 8 Division of Infectious Diseases, Northwestern University Feinberg School of Medicine, Chicago, Illinois, United States of America; 9 Infectious Diseases and Microbiology, Graduate School of Public Health, University of Pittsburgh, Pittsburgh, Pennsylvania, United States of America; 10 Department of Medicine, David Geffen School of Medicine, University of California–Los Angeles, Los Angeles, California, United States of America; 11 Department of Molecular Microbiology and Immunology, Johns Hopkins Bloomberg School of Public Health, Baltimore, Maryland, United States of America; 12 Cancer and Inflammation Program, Laboratory of Experimental Immunology, SAIC-Frederick, Inc., NCI-Frederick, Frederick, Maryland, United States of America; 13 Nuffield Department of Clinical Medicine, University of Oxford and Weatherall Institute of Molecular Medicine, John Radcliffe Hospital, Headington, Oxford, United Kingdom; 14 Division of Viral Pathogenesis, Beth Israel Deaconess Medical Center, Harvard Medical School, Boston, Massachusetts, United States of America; 15 Medical Research Council Human Immunology Unit, Weatherall Institute of Molecular Medicine, John Radcliffe Hospital, Oxford, United Kingdom; 16 Duke Human Vaccine Institute, Duke University, Durham, North Carolina, United States of America; 17 Institute of Microbiology, University Hospital Center; and University of Lausanne, Lausanne, Switzerland; 18 Ragon Institute of MGH, MIT and Harvard, Boston, Massachusetts, United States of America; Fred Hutchinson Cancer Research Center, United States of America

## Abstract

A genome-wide screen for large structural variants showed that a copy number variant (CNV) in the region encoding killer cell immunoglobulin-like receptors (KIR) associates with HIV-1 control as measured by plasma viral load at set point in individuals of European ancestry. This CNV encompasses the *KIR3DL1-KIR3DS1* locus, encoding receptors that interact with specific HLA-Bw4 molecules to regulate the activation of lymphocyte subsets including natural killer (NK) cells. We quantified the number of copies of *KIR3DS1* and *KIR3DL1* in a large HIV-1 positive cohort, and showed that an increase in *KIR3DS1* count associates with a lower viral set point if its putative ligand is present (*p* = 0.00028), as does an increase in *KIR3DL1* count in the presence of *KIR3DS1* and appropriate ligands for both receptors (*p* = 0.0015). We further provide functional data that demonstrate that NK cells from individuals with multiple copies of KIR3DL1, in the presence of KIR3DS1 and the appropriate ligands, inhibit HIV-1 replication more robustly, and associated with a significant expansion in the frequency of KIR3DS1+, but not KIR3DL1+, NK cells in their peripheral blood. Our results suggest that the relative amounts of these activating and inhibitory KIR play a role in regulating the peripheral expansion of highly antiviral KIR3DS1+ NK cells, which may determine differences in HIV-1 control following infection.

## Abstract 2

The authors that the number of activating and inhibitory *KIR* genes varies between individuals and plays a role in the regulation of immune mechanisms that determine HIV-1 control.

## Author Summary

There is marked intrinsic variation in the extent to which individuals are able to control HIV-1. We have identified a genetic copy number variable region (CNV) in humans that plays a significant role in the control of HIV-1. This CNV is located in the genomic region that encodes the killer cell immunoglobulin-like receptors (KIRs) and specifically affects the *KIR3DS1* and *KIR3DL1* genes, encoding two KIRs that interact with human leukocyte antigen B (HLA-B) ligands. KIRs are expressed on the surface of natural killer (NK) cells, which serve as important players in the innate immune response, and are involved in the recognition of infected and malignant cells through a loss or alteration in “self” ligands. We use both genetic association and functional evidence to show a strong interaction between KIR3DL1 and KIR3DS1, indicating that increasing gene counts for *KIR3DL1* confer increasing levels of protection against HIV-1, but only in the presence of at least one copy of *KIR3DS1*. This effect was associated with a dramatic increase in the abundance of KIR3DS1+ NK cells in the peripheral blood, and strongly associated with a more robust capacity of peripheral NK cells to suppress HIV-1 replication in vitro. This work provides one of the few examples of an association between a relatively common CNV and a human complex trait.

## Introduction

**Figure 1:**
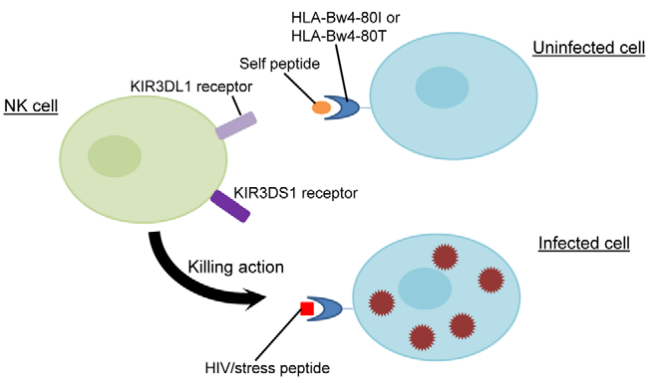
KIR and HLA-B interactions. An NK cell can express KIR3DL1 alone, KIR3DS1 alone, both receptors, or neither. KIR3DL1 is an inhibitory receptor and has been shown to interact with HLA-Bw4-80I and with HLA-Bw4-80T. KIR3DS1 is an activating receptor and may interact with HLA-Bw4-80I, possibly through an indirect mechanism. An NK cell can be activated to lyse an infected cell via either the activating of KIR receptor signaling or the dampening of inhibitory KIR activity.

The KIR receptors are expressed mainly on the surface of lymphocyte subsets including natural killer (NK) cells and a small subset of T cells, and they have a unique role in fine-tuning the balance between self-tolerance and cytotoxicity. KIRs bind to major histocompatibility complex (MHC) class I ligands on the surface of target cells. The degree of inhibition and/or activation mediated by interactions between co-inherited KIR and MHC class I gene products determines the activation threshold for NK cells (Figure 1) [1].

The KIR region, on chromosome 19q13.4, is highly polymorphic in humans [2] and its extensive polymorphism has been repeatedly associated with the natural history of HIV-1 infection [3]. The *KIR3DL1* and *KIR3DS1* genes segregate as allelic variants at the same locus and both are thought to encode receptors for molecules that fall within the Bw4 subfamily of *HLA-B* alleles (HLA-Bw4). There is also evidence for a single chromosome to have both *KIR3DS1* and *KIR3DL1* [4],[5].

Both allele groups at this locus have been shown to be involved in HIV-1 pathogenesis. The activating allele *KIR3DS1*, in combination with HLA-Bw4 molecules that have an isoleucine at position 80 (Bw4-80I), has been associated with lower viral load, slower decline in CD4+ T cells, and delayed progression to AIDS, as well as with protection against opportunistic infections [6],[7]. In addition, *KIR3DS1* has recently been shown to correlate with strong inhibition of HIV-1 replication [8]. However, some reports have shown no protective effect associated with the *KIR3DS1*+*HLA-Bw4-80I* genotype [9],[10] or show no evidence for a synergistic effect from the *KIR3DS1*+*HLA-Bw4-80I* genotype on viral load or on CD4+ T cell counts [11]. Recent reports have also shown that NK cells expressing KIR also directly place pressure of the virus, driving HIV viral evolution [12].

Similarly, various distinct allelic combinations of the inhibitory KIR3DL1 receptor and HLA-Bw4 ligands have been associated with lower HIV-1 viral load and slower progression to AIDS [13]. Two proposed functional explanations may account for the latter result. The first relates to the education process of NK cells during development, in which inhibitory receptors must recognize autologous MHC class I ligands for the NK cell to be functional upon maturation [14]–[16], suggesting that ligand engagement by more highly expressed inhibitory KIR3DL1 allotypes during NK cell development ultimately may result in stronger NK cell responses in the event of viral infection when the ligand is missing or altered [14],[17]. The second underlying explanation may relate to the fact that KIR3DL1 is involved in monitoring the circulation for normal MHC class I expression; however upon HIV infection, HIV Nef protein rapidly downregulates MHC class I expression. Thus, it is equally plausible that higher expression of KIR3DL1 may allow NK cells to recognize reduced MHC class I expression on infected cells more readily.

KIR receptors are expressed on NK cells in a variegated manner, where only a fraction of all NK cells express a particular KIR gene product. Certain KIR receptors are consistently expressed on a large fraction of NK cells, while others are expressed on a smaller fraction of NK cells [18]. In individuals with one copy of *KIR3DS1*, roughly 20%–50% of NK cells express the KIR3DS1 receptor [18],[19], and in individuals with two copies of *KIR3DS1*, 60% or greater of NK cells express the KIR3DS1 receptor [18],[19]. To add to the complexity, some *KIR3DL1* allotypes have different surface expression levels [20], which have been shown to have varying impacts on HIV-1 outcomes [13], and correlate with genealogical groups of *KIR3DL1* alleles [21].

The primary outcome studied here is HIV-1 viral load at set point, which has been shown to be a genetically tractable HIV outcome [22]. We used a genome-wide screen to identify a copy number variable region that associated with HIV-1 control, as measured by plasma viral load at set point, and that encompassed the *KIR3DL1-KIR3DS1* locus. Further dissection of the region and of the interactions between KIR3DL1, KIR3DS1, and their HLA ligands demonstrated that the number of gene copies of the inhibitory KIR3DL1 receptor and activating KIR3DS1 receptor plays an important role in modulating HIV-1 control, but that this effect is only detectable after epistatic interactions between HLA molecules and KIR receptors are taken into account. Furthermore, functional and transcriptional studies on cells derived from individuals with these particular KIR CNV/HLA combinations demonstrated a dramatic expansion of KIR3DS1+ NK cells, which are able to robustly inhibit HIV replication in vitro. Thus, these data support the genetic association results, suggesting novel mechanisms of regulation of the antiviral activity of NK cells.

## Results

### CNV Identification in KIR Region

We investigated the role of large CNVs on HIV-1 control in a cohort of 2,102 patients of European ancestry from the Euro-CHAVI Consortium and the Multicenter AIDS Cohort Study (MACS). Genome-wide single nucleotide polymorphism (SNP) genotyping was performed using Illumina's HumanHap550, Human1M, or Human1MDuo BeadChips, and we used the PennCNV software [23] to identify large CNVs. For each SNP in a CNV region, we assigned copy number status (zero, one, two, three, or four copies) to each sample and carried out a linear regression analysis on HIV-1 viral load at set point. We examined duplications and deletions separately using copy number status in additive genetic models and included as covariates age, sex, and 12 EIGENSTRAT ancestry axes to control for population stratification [24]. We tested 5,384 deletions and 3,553 duplications with a minimum frequency of 0.004, and none of them associated significantly with viral set point after correction for multiple testing using straight Bonferroni correction (*p threshold* = 9.2×10^−6^ for deletions and 1.4×10^−5^ for duplications). However, we note that this is a conservative correction, because several CNVs can reflect the same association signal due to the difficulty of distinguishing between nearby CNVs when inferring them from the genotyping data.

We manually inspected all CNVs that showed an association with set point at *p*<0.05 (unadjusted) (Table S1). One associated CNV was located in the KIR region, where both duplications and deletions associated to variable degrees with HIV-1 control. The duplications and deletions each occurred in around 3%–5% of the study population. Many, although not all, of these identified duplications and deletions covered the *KIR3DL1-KIR3DS1* locus, which has been the subject of intensive study related to control of HIV-1 [7],[13]. Focusing on SNPs included in this copy number variable region (rs631717, rs649216, rs581623), HIV-1 viral load at set point was lower for individuals with more copies and higher for individuals with fewer copies (*p* = 0.010 for duplications and *p* = 0.001 for deletions, as compared to samples that did not show copy number variability and have two total copies of *KIR3DL1* and/or *KIR3DS1*). If we assign an overall copy number to each sample based on the PennCNV call for these three SNPs (zero, one, two, three, or four copies), the CNV in the KIR region shows an even stronger association with viral load at set point (*p* = 3×10^−5^).

### *KIR3DL1* and *KIR3DS1* Gene Counts

To assess the individual impact of *KIR3DS1* and *KIR3DL1* on HIV-1 control and to further investigate the copy number variability observed in the KIR region, we developed a real-time PCR assay to quantify the number of copies of each gene (Figure S1). Individuals without a CNV in the region have a total of two copies of these genes (one *KIR3DL1* or one *KIR3DS1* on each chromosome), whereas in individuals with a deletion or a duplication their sum corresponds to the copy number state (e.g., four alleles are measured in individuals with a homozygous duplication, or zero alleles with a homozygous deletion) (Table S2). As can be seen in Figure S1, our assay is able to count nearly all known alleles of *KIR3DS1/KIR3DL1* that appear in populations of European descent. Overall, we found high repeatability of the assay (Figure S2) and a good correspondence with copy number assignments called by PennCNV [23] using SNPs in the *KIR3DL1-KIR3DS1* region (Text S1, Table S3).

In the *KIR3DL1-KIR3DS1* region, about 3.6% of our samples had a deletion according to the real-time PCR data, and about 5% had a duplication. The frequencies of the various genotypes that show evidence of duplication are listed in Table S4. It is clear that a single chromosome can have two copies of *KIR3DS1* or *KIR3DL1*, since respectively 6% and 14.5% of the samples with duplications had three total copies of either *KIR3DS1* or *KIR3DL1*, presumably two on one chromosome and one on the opposite chromosome.

The diversity of genotypes present at this locus and our inability to discern which genes occur on the same chromosome make it challenging to determine if this locus is in Hardy-Weinberg equilibrium. However, previous work has shown that *KIR3DL1/KIR3DS1* are indeed in Hardy-Weinberg equilibrium, in spite of the existence of the relatively rare haplotypes containing multiple copies of this gene [25].

### Raw Counts of *KIR3DS1* and *KIR3DL1* Do Not Associate with Set Point

We first checked to see if the raw number of *KIR3DS1* and *KIR3DL1* copies per individual (without accounting for presence of the cognate HLA-B ligand) associated with HIV-1 set point: neither raw count associated with viral control (*n* = 1,736, *p* = 0.230 for raw *KIR3DS1* count and *p* = 0.508 for raw *KIR3DL1* count, Table 1).

**Table 1:** *p*-Values for association with VL set point.

	Alone^a^ (*n* = 1,736)	Alone^a^ (*n* = 706)
	*p*	*p*
Raw *KIR3DS1* count	0.230	0.254
Raw *KIR3DL1* count	0.508	0.768

a Model includes age, gender, 12 EIGENSTRAT axes, and either *KIR3DL1* or *KIR3DS1* raw count.

The first column shows the *p* values for the association between all samples with a raw *KIR3DL1* and a raw *KIR3DS1* count, and the second column is limited to just the 706 samples that are used in Table 3.

### Effective Gene Count

The functionality of a KIR receptor hinges on the presence of its cognate ligand. The activity of KIR3DS1 and KIR3DL1 therefore depends on the expression of appropriate HLA-Bw4 molecules on the surface of target cells. To take this epistatic feature into account, we created an “effective” gene count, in which each copy of *KIR3DL1* or *KIR3DS1* was counted only when its specific Bw4 ligand was present. HLA-Bw4-80I and HLA-Bw4-80T have both been demonstrated to be ligands for KIR3DL1 [26],[27]. Although the direct interaction between KIR3DS1 and HLA-Bw4-80I is less definitive because a physical interaction between HLA-Bw4-80I and KIR3DS1 has not been demonstrated [28]–[30], epidemiological and functional evidence suggests that under some conditions, such as HIV-1 infection, HLA-Bw4-80I serves directly or indirectly as a ligand for KIR3DS1 (Table 2) [3]. HLA-Bw6 is not a ligand for either KIR3DS1 or KIR3DL1, and there has not been evidence to show that any other KIR receptors interact with HLA-Bw4 molecules. Some HLA-A alleles also carry the HLA-Bw4-80I motif (16.1% of all HLA-A in dbMHC Project Anthropology, http://www.ncbi.nlm.nih.gov/gv/mhc/ihwg.cgi?ID=9&cmd=PRJOV), but for simplicity we restricted our analyses to HLA-B alleles with Bw4-80I, since it is not clear that all HLA-A-Bw4-80I molecules serve as ligands for KIR3DL1. All subsequent analyses used this “effective” gene count.

**Table 2:** Combinations of KIR3DS1-KIR3DL1 and HLA-B treated as receptor/ligand pairs in this study.

	HLA-Bw4 80I	HLA-Bw4 80T	HLA-Bw6
KIR3DS1	Y	N	N
KIR3DL1	Y	Y	N

In order to determine these “effective” KIR3DL1 and KIR3DS1 counts, we required that samples had (1) successful real-time quantitation for both *KIR3DL1* and *KIR3DS1*, (2) *HLA-B* data, and (3) available *KIR3DL1* allelic subtyping, if at least one *KIR3DL1* was present. A total of 706 samples fit all the criteria. Allelic subtyping was not included for *KIR3DS1* since it shows little variation [21]. The *KIR3DL1* subtyping data were used to separate the alleles that are expressed on the cell surface (“KIR3DL1-surface”), at either high or low levels, from the special case of KIR3DL1*004, which is not expressed at the cell surface [13],[31].

### Effective Counts of KIR3DS1 and KIR3DL1 Associate with Set Point

**Figure 2:**
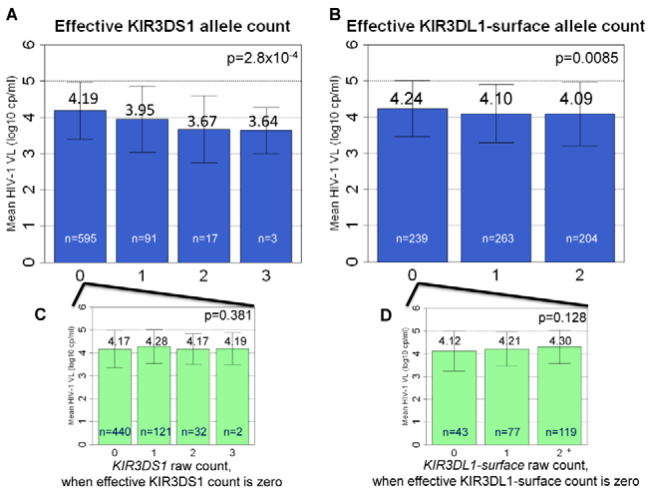
Association between KIR3DS1 and KIR3DL1 effective counts and viral load at set point. (A–B) The effective counts of KIR3DS1 and KIR3DL1-surface associate with viral load (VL) at set point. (C–D) The insets show the association between the raw *KIR3DS1* or *KIR3DL1-surface* count, for the subset of patients from graphs A and B where the effective counts equal zero. An effective count of zero can be due to the absence of the KIR receptor or due to the absence of the HLA-Bw4 ligand. The raw count does not associate with viral load at set point when the effective count for KIR3DS1 or KIR3DL1-surface equals zero. Error bars show one standard deviation.

Each of the effective counts was tested separately. We found that the effective KIR3DS1 and effective KIR3DL1-surface gene counts associated with HIV-1 set point (*p* = 4.2×10^−6^ and 0.020, respectively) (Table 3), with an increase in effective gene count leading to lower viral loads. When KIR3DS1 and KIR3DL1-surface effective gene counts were considered in the same regression model, they remained separately significant (*p* = 0.00028 and 0.0085, respectively) (Table 3, Figure 2A–B).

**Table 3:** *p*-Values for association with VL set point.

		Alone	In Combined	Model	In Combined	Model
	*n*	*p*	*n*	*p*	*n*	*p*
Effective KIR3DS1 count	1,429	4.2E-06^a^	706	2.8E-04^b^	706	0.0075^c^
Effective KIR3DL1-surface count	749	0.020^a^	706	0.0085^b^	706	0.220^c^
HLA-B*57	*-----*	*-----*	*-----*	*-----*	*706*	*1.4E-07* c
HLA-B*27	*-----*	*-----*	*-----*	*-----*	*706*	*0.0049* c
HLA-B*35Px	*-----*	*-----*	*-----*	*-----*	*706*	*0.098* c

a Model includes age, gender, 12 EIGENSTRAT axes, and the count of one KIR gene.

b Model includes age, gender, 12 EIGENSTRAT axes, effective KIR3DS1 count, and effective KIR3DL1-surface count.

c Model includes age, gender, 12 EIGENSTRAT axes, effective KIR3DS1 count, effective KIR3DL1-surface count, B*5701, B*27, and B*35Px.

**Figure 3:**
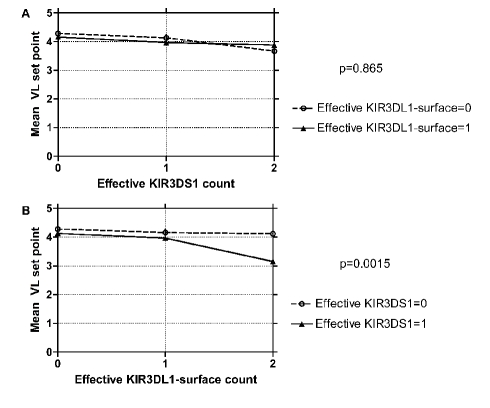
Interaction between KIR3DL1 and KIR3DS1. (A) Test of whether KIR3DL1 status influences the effect of KIR3DS1 on viral load. (B) Test of whether KIR3DS1 status influences the effect of KIR3DL1 on viral load.

Regardless of KIR3DL1 status, an increase in the effective count of KIR3DS1 associated with improved viral control (Table 4, Figure 3A). In contrast, an increase in the effective count of KIR3DL1 did not show any association in the absence of KIR3DS1, but did impact HIV-1 set point in the presence of one or more effective copies of KIR3DS1 (*p* = 0.0015, Table 4, Figure 3B).

**Table 4:** Interaction between KIR3DS1 and KIR3DL1-surface.

	Effective KIR3DL1- Surface Count = 0^b^		Effective KIR3DL1- Surface Count = 1^b^		Effective KIR3DL1- Surface Count = 2	
	*n*	MeanSet Point	*n*	MeanSet Point	*n*	MeanSet Point
Effective KIR3DS1 count = 0^a^	211	4.28	186	4.16	198	4.12
Effective KIR3DS1 count = 1^a^	14	4.13	72	3.97	5	3.15
Effective KIR3DS1 count = 2+	14	3.67	5	3.88	1	2.53

a *p* = 0.0015. Comparison tests whether KIR3DS1 status influences the effect of KIR3DL1 on viral load.

b *p* = 0.865. Comparison tests whether KIR3DL1 status influences the effect of KIR3DS1 on viral load.

In the subset of study participants who had two *HLA-B* alleles from the Bw6 subfamily (Bw6/Bw6 homozygotes), the raw counts for *KIR3DS1* and *KIR3DL1-surface* showed no association with HIV-1 viral load at set point, further supporting the critical nature of particular KIR-HLA combined genotypes (Table S5).

### KIR3DS1 Effective Count Associates with Set Point When HLA-B*57, HLA-B*27, and HLA-B*35Px Are Included as Covariates in Model

The interpretation of KIR-HLA epistatic influences on HIV-1 control is complicated by the fact that particular *HLA* class I alleles (*-A*, *-B*, and -*C*) have previously been independently implicated in modulating HIV-1 control, with *HLA-B* alleles placing the greatest immune pressure on HIV-1 replication [32]. Specifically, HLA-B*57 and HLA-B*27 are associated with slower HIV-1 disease progression, whereas HLA-B*35Px associates with a stronger susceptibility to developing AIDS rapidly [33]–[35]. The differential impact of these HLA alleles has been attributed to differences in epitope presentation in conserved versus variable regions within the viral genome. The protective HLA-B*57 and HLA-B*27 generate strong antiviral CD8+ T cell responses that target highly conserved proteins, where escape mutations place a great impact on viral fitness. In contrast, HLA-B*35Px restricted CD8+ T cells tend to target highly variable regions that likely place little pressure on the virus. However, in addition to CD8+ T cells, NK cells are also able to interact differentially with these three alleles via KIR3DL1 and KIR3DS1. HLA-B*57 molecules are a subset of the HLA-Bw4-80I group (ligand for KIR3DL1 and possibly KIR3DS1), HLA-B*27 are mainly HLA-Bw4-80T (ligand for KIR3DL1), and HLA-B*35Px are primarily HLA-Bw6 (except HLA-B*5301, which is HLA-Bw4-80I) (Table S6). Thus, although HLA-B*27 and HLA-B*57 may themselves interact with KIR, these favorable and unfavorable HLA alleles (in terms of HIV-1 control) are present in different proportions within each of the groups of alleles that are and are not ligands for KIR3DL1 and KIR3DS1, and could therefore drive an association with viral control independently of KIR genotypes.

For this reason, to assess the specific effects of KIR, these *HLA-B* alleles must be accounted for in the analysis. To do this, we added all three *HLA-B* allotypes as covariates to the model considered earlier. We found that an increase in the effective count of KIR3DS1 still associated with a decrease in viral load (*p* = 0.0075), but the effective count of KIR3DL1-surface did not (*p* = 0.220) (Table 3).

We note that correction for these controlling alleles could reduce power and create instabilities in the signal due to colinearity. Nevertheless, we retain this test as one established approach [4] for evaluating whether it appears to be a credible alternative explanation that the apparent signal is due simply to the contribution of these controlling alleles. We also note that some fraction of the protection conferred by HLA-B*57 and HLA-B*27 is due to their interaction with KIR receptors [13].

### Higher Effective Counts for Co-Expressed KIR3DL1 and KIR3DS1 Are Associated with the Generation of NK Cells with a Superior Capacity to Inhibit HIV-1 Replication In Vitro

Previous work examining the role of protective KIR/HLA genotypes on NK cell functionality showed that NK cells from healthy HIV-uninfected individuals who expressed KIR3DS1 and that also expressed HLA-Bw4-80I were associated with a robust capacity to inhibit HIV-1 replication in vitro, compared to individuals who expressed KIR3DS1 in the absence of its putative ligand [8], potentially conferring an enhanced capacity of these individuals to respond to the virus soon after infection. We were therefore interested in determining whether individuals with the observed duplication showed a differential capacity to inhibit viral replication in vitro, and whether this effect was due to KIR3DS1, KIR3DL1, or both. To determine whether NK cells generated in individuals with increased effective counts of KIR3DS1 and KIR3DL1 showed any variation in NK cell function, we performed an NK cell viral inhibition assay using fresh blood collected from HIV-negative individuals with different KIR/HLA genotype combinations, including several individuals with one effective copy of KIR3DS1 and two effective copies of KIR3DL1.

**Figure 4:**
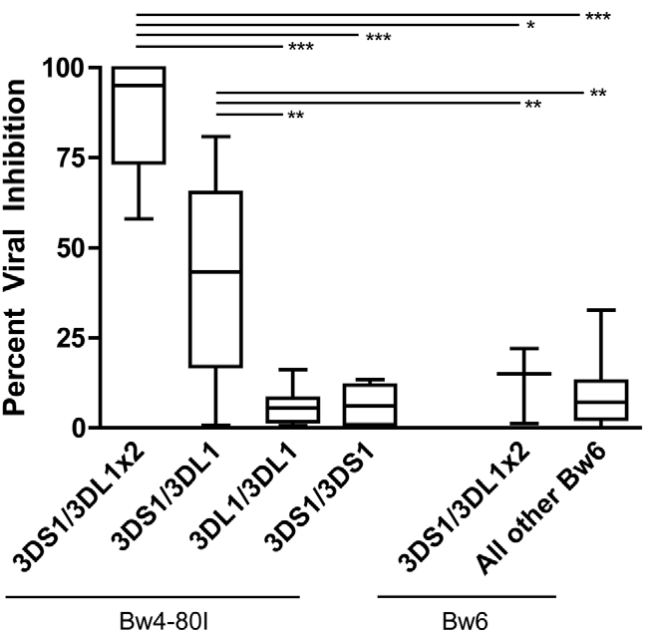
NK cell inhibition of HIV-1 replication in vitro. The inhibitory capacity of NK cells from HIV-negative donors with different combinations of KIR3DL1 and/or KIR3DS1 was tested in an NK cell inhibition assay. NK cells derived from individuals who express a KIR3DS1 and two KIR3DL1 exhibit a remarkably robust capacity to inhibit HIV-1 replication in vitro if HLA-Bw4-80I is present (mean inhibition = 88%, *n* = 5), but not if HLA-Bw4-80I is absent (mean inhibition = 13%, *n* = 3) (*p*<0.05). Similarly, NK cells derived from individuals who express one KIR3DL1, one KIR3DS1, and HLA-Bw4-80I also inhibit HIV-1 replication (mean inhibition = 42%, *n* = 19) much better than individuals with HLA-Bw4-80I and two KIR3DL1 (mean inhibition = 6%, *n* = 10), HLA-Bw4-80I and two KIR3DS1 (mean inhibition = 6%, *n* = 4), or individuals who do not have HLA-Bw4-80I (mean inhibition = 8%, *n* = 35) (*p*<0.005) (* *p*<0.05, ** *p*<0.005, *** *p*<0.0005).

We found that NK cells from HIV-negative individuals with one effective copy of KIR3DS1 and one effective copy of KIR3DL1 inhibited HIV-1 replication more potently than NK cells from individuals who did not possess at least one effective copy of both KIR3DL1 and KIR3DS1 (Figure 4, mean inhibition = 42%, *p*<0.005). Interestingly, individuals who had one effective copy of KIR3DS1 and two effective copies of KIR3DL1 exhibited even more robust NK-cell-mediated inhibition of HIV replication in vitro than did individuals who had one copy of effective KIR3DS1 and just one copy of effective KIR3DL1 (Figure 4, mean inhibition = 88%). Individuals who did not have HLA-Bw4-80I or who did not have both KIR3DL1 and KIR3DS1 showed markedly less inhibition (mean inhibition<15%). These data support the association results described in the first part of the article, with the only discrepancy being that individuals with two effective copies of KIR3DS1 do show a decrease in viral load at set point, but do not show an increase in viral inhibition. Overall, these results demonstrate that, prior to infection, NK cells generated in the presence of more effective copies of KIR3DS1 and KIR3DL1 have enhanced HIV-1 antiviral activity.

### Individuals with Effective Copies of KIR3DS1 and KIR3DL1 Show an Elevated Frequency of KIR3DS1+ NK Cells and Elevated KIR3DS1 Transcript Levels as the Number of Copies of KIR3DL1 Increases

Mounting evidence suggests that KIR/HLA compound genotypes heavily influence the frequency of NK cells expressing a given KIR receptor [36],[37], and it has previously been shown that KIR3DS1+ NK cells expand in acute HIV infection in the presence of their putative ligand, HLA-Bw4-80I [38]. This preferential early expansion of highly antiviral KIR3DS1+ NK cells could potentially provide enhanced viral control. Given that increased effective counts of KIR3DL1 in the presence of KIR3DS1 demonstrated an enhanced capacity to inhibit HIV replication (Figure 4), we speculated that the increasing doses of inhibitory KIRs may be associated with unique patterns of KIR3DL1 and KIR3DS1 expression levels in NK cells. This could potentially account for their superior antiviral activity.

**Figure 5:**
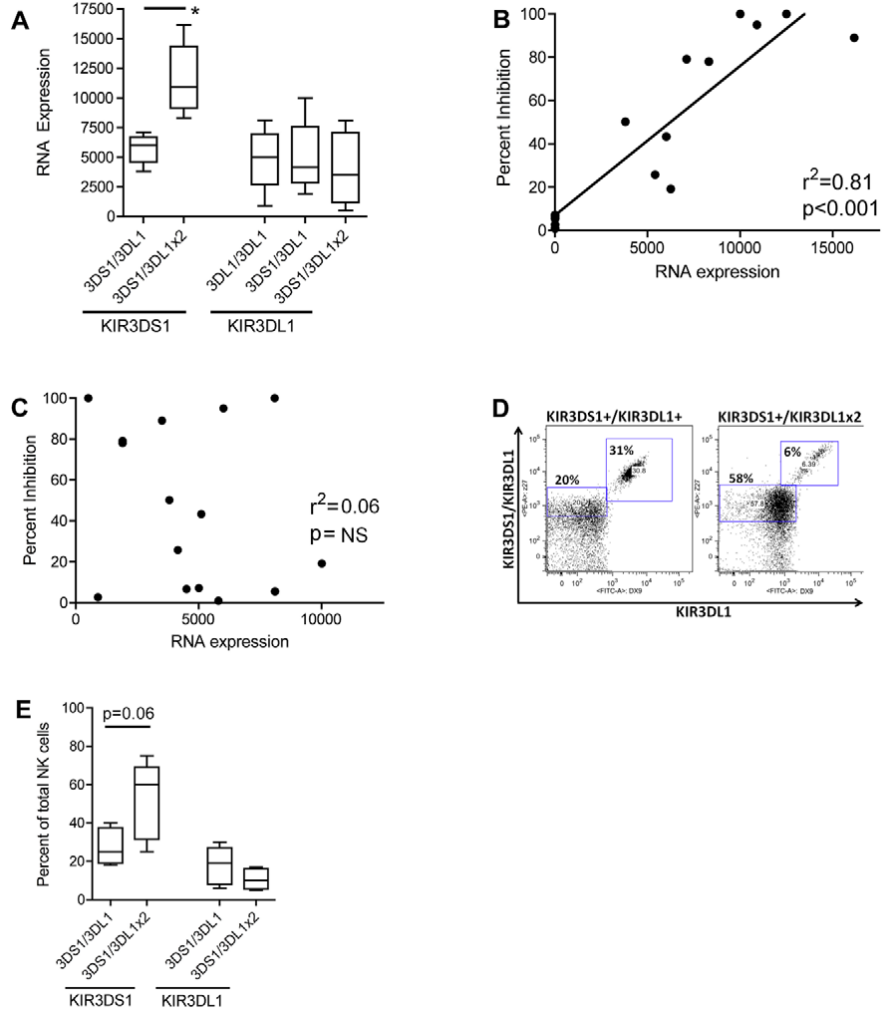
Expression patterns of effective KIR3DS1 and effective KIR3DL1. Increasing the copies of effective KIR3DL1 in the presence of effective KIR3DS1 results in elevated KIR3DS1 transcript levels, and an increased frequency of NK cells expressing KIR3DS1 in the peripheral blood. (A) The levels of KIR3DS1 and KIR3DL1 transcripts were assessed by quantitative PCR analysis in purified populations of NK cells, demonstrating that individuals who express increasing copies of effective KIR3DL1 in the presence of an effective KIR3DS1 possess increasing amounts of KIR3DS1 transcripts, but not KIR3DL1 transcripts (*n* = 5 in each group). The level of KIR3DS1 transcripts (B) but not KIR3DL1 (C) correlates with the level of in vitro NK-cell-mediated inhibition. (D) Furthermore, raw flow cytometric data from two representative individuals show that the protective genotype of one effective KIR3DS1 and two effective KIR3DL1 is associated with an elevated frequency of KIR3DS1+ NK cells (58%) in the peripheral blood. Z27, on the *y*-axis, stains for both KIR3DS1 and KIR3DL1, and DX9 on the *x*-axis stains for just KIR3DL1. (E) These same individuals show a trend towards an accumulation of KIR3DS1+ NK cells, but not KIR3DL1+ NK cells, in the peripheral circulation compared to individuals who have fewer copies of KIR3DL1 in the presence of an effective KIR3DS1 gene count (*n* = 5 in each group) (* *p*<0.05).

Using freshly isolated purified NK cells from healthy controls with distinct KIR/HLA genotypes, we found that increasing effective counts of KIR3DL1, in the presence of an effective KIR3DS1, were associated with an elevated number of KIR3DS1 transcripts in purified NK cell populations (Figure 5A, *p*<0.05). This suggests that the increasing effective KIR3DL1 counts potentiate the expression of KIR3DS1 in the circulating NK cell pool but have little impact on their own expression. More interestingly, increasing levels of KIR3DS1 RNA transcripts were strongly associated with the level of HIV-1 inhibition in all KIR3DS1-carrying individuals expressing the putative ligand (r^2^ = 0.81, *p*<0.001, Figure 5B), whereas the relative expression of KIR3DL1 was not associated with NK-cell-mediated inhibition of HIV infection (Figure 5C). Similarly, in addition to the impact of increasing KIR3DL1 effective counts on KIR3DS1 transcript expression, individuals with the protective genotype of an effective copy of KIR3DS1 and two effective copies of KIR3DL1 showed a trend towards an expansion of KIR3DS1+ NK cells in the peripheral circulation, as compared to individuals with a single effective copy of KIR3DL1 in the presence of an effective copy of KIR3DS1 (Figure 5D,E). The shift in the whole NK cell population (Figure 5D) could reflect an increase in the quantity of KIR3DS1 expressed on the surface of NK cells in the presence of two copies of KIR3DL1, concomitant with an expansion of the frequency of KIR3DS1+ NK cells [39]. Individuals with the protective genotype of an effective copy of KIR3DS1 and two effective copies of KIR3DL1 also show an increase in the percent of KIR3DS1+ NK cells, but not an increase in the percent of KIR3DL1+ NK cells, when compared to individuals with just one effective copy of KIR3DS1 and one effective copy of KIR3DL1 (Figure 5E). These data suggest that the increasing KIR3DL1 effective gene count, in the presence of an effective KIR3DS1, is associated with more robust HIV antiviral activity due to a genotype-driven natural expansion of KIR3DS1+ NK cells in the peripheral blood prior to infection. This population of KIR3DS1+ NK cells may expand even further upon HIV infection, potentially providing these individuals with an antiviral advantage.

## Discussion

We conducted a genome-wide CNV screen that allowed us to locate a variable region involved in HIV-1 control. The observed association signal was due to *KIR3DL1* and *KIR3DS1* copy number variation encompassed within the region, and to the interaction of these receptors with their cognate HLA-B ligands. The proportion of variance explained by the CNV and the proportion of variance explained by the effective KIR3DL1 and KIR3DS1 counts are both approximately equal to 0.7% (in models that also include age, gender, 12 significant EIGENSTRAT axes, HLA-B*57, HLA-B*27, and HLA-B*35Px). This compares favorably with the proportion of variance explained by CCR5 (1.7%) and CCR2 (1%) and is substantially lower than that explained by HLA-B*57 (5.8%) [40].

The effective count model that we developed uses one term to describe the interaction between KIR molecules and their known or suggested HLA-B ligands, under the assumption that a receptor is not functional unless its HLA-B ligand is present. The model is based on the well-established interaction between KIR3DL1 and HLA-Bw4, and the possible interaction between KIR3DS1 and HLA-Bw4-80I. Our results actually further support the proposed epistatic interaction between KIR3DS1 and HLA-Bw4-80I, as can be seen in the striking difference in viral inhibition exhibited by KIR3DS1+ cells from KIR3DL1+ individuals who do and do not have HLA-Bw4-80I.

HLA class I alleles are key players in the adaptive immune response, having marked differences in their abilities to restrict HIV through presentation of diverse HIV epitopes to cytotoxic T lymphocytes (CTL), which will in turn kill the infected cells. But HLA molecules also interact with KIR to modulate NK cells, thereby acting within the innate arm of the immune response. In fact, the three alleles described above also are subsets of the HLA-Bw4-80I (for HLA-B*57), the HLA-Bw4-80T (for HLA-B*27), and the HLA-Bw6 groups (for HLA-B*35Px). Their impact on HIV control through T-cell-mediated immunity is therefore also measured in the global assessment of the effect of the KIR3DS1 and KIR3DL1 effective counts. We confirmed that our results, showing an association between a decrease in viral load at set point and an increase in the effective KIR3DS1 and KIR3DL1 counts, were not merely due to this confounding factor by including the relevant *HLA* alleles as covariates in combined models. As expected, the effective KIR3DS1 and KIR3DL1 association signals were weaker after adjustment for the *HLA* alleles, but the effective KIR3DS1 count remained significantly associated with viral load, and there is evidence for a KIR3DL1 effect when KIR3DS1 is present. Indeed, part of the protective effect of HLA-B*57/HLA-B*27 and the susceptibility effect of HLA-B*35Px are likely attributable to their interaction (or lack thereof) with KIR3DL1 and/or KIR3DS1 [13]. Thus, these interactions between KIR and HLA provide an additional contribution to HIV-1 control.

Increasing copies of effective KIR3DS1 had a clear impact on viral load at set point regardless of the presence of an effective KIR3DL1 and even after accounting for *HLA-B* controlling alleles. However, NK cells derived from individuals with multiple copies of effective KIR3DS1 in the absence of an effective KIR3DL1 did not appear to mediate robust antiviral activity in vitro. This is potentially due to the fact that this assay may not detect the antiviral activity of these cells, as these cells may recognize or respond to target cells in a distinct manner than NK cells from individuals who co-express KIR3DL1. Or this discrepancy could be related to the manner in which KIR3DS1 and KIR3DL1 recognize, compete, and interact with the same ligands on infected cells.

Increasing effective copies of KIR3DL1 in the absence of an effective KIR3DS1 had no impact on viral control after correcting for known protective *HLA-B* alleles. Individuals without an effective KIR3DS1 also did not show an increase in the capacity to inhibit HIV-1 replication in vitro.

However, individuals who had additional copies of effective KIR3DL1 in addition to at least one effective copy of KIR3DS1 exhibited remarkable control of HIV-1 set point viral loads. Moreover, this finding was supported by our functional data, where individuals with effective KIR3DS1 and KIR3DL1 also showed an elevated capacity to inhibit HIV replication in vitro. Interestingly, individuals with two effective copies of KIR3DL1 and an effective copy of KIR3DS1 showed even more inhibition than individuals with just one copy of each gene. They also showed a significant elevation in KIR3DS1 transcript levels and in KIR3DS1+ NK cells expressing this activating KIR receptor as compared to individuals with just one effective copy of KIR3DS1 and one effective copy of KIR3DL1 (Figures 4, 5A and 5E). Surprisingly, having additional effective copies of KIR3DL1 also increased the proportion of KIR3DS1+ NK cells. To our knowledge, this is the first study to show evidence for a beneficial interaction between KIR3DS1 and KIR3DL1, and these data imply that an elevated KIR3DL1 effective count may specifically provide more robust licensing of KIR3DS1+ NK cells that are then able to expand and mediate strong antiviral control. Alternatively, HIV proteins such as Nef [41] and Vpu [42]–[44] specifically interfere with the capacity of NK cells to recognize infected cells via the downregulation of various NK cell receptor ligands. Thus, it is equally possible that increased expression of KIR3DL1 may provide a more sensitive measure of reduced MHC class I expression, potentiating the triggering of other activating NK cell receptors.

Previous data suggest that individuals with increasing copies of KIR3DS1 exhibit an expansion of the frequency of KIR3DS1+ NK cells in their peripheral circulation [39]. However, such patterns have not been observed for inhibitory KIR such as KIR3DL1, perhaps due to the fact that there are many unique KIR3DL1 alleles, which can be grouped into high, low, and unexpressed variants. Thus, increasing the dosage of KIR3DL1 may alter NK cell functionality even though it does not necessarily increase the frequency of KIR3DL+NK cells. Perhaps having additional copies of KIR3DL1 could contribute to enhanced licensing, similar to the manner in which additional copies of HLA-Bw4 enhance bulk NK cell activity [45]. However, additional work is required to tease out the mechanism underlying the potential interaction between KIR receptors.

The analyses that we have conducted required several types of data (genome-wide genotyping, real-time quantitation for *KIR3DL1* and *KIR3DS1*, *HLA-B* allelic determination, *KIR3DL1* subtyping), which limited our final sample size. However, the results of our association studies appear clear and biologically reasonable, and are strongly supported by functional data, providing a plausible mechanism by which this CNV may impact HIV-1 control.

KIR receptors are expressed on NK cells in a stochastic manner and are involved in modulating NK cell functions. The CNV that we have observed in the KIR region can influence the proportion of NK cells expressing KIR3DS1, and possibly the overall expression level of KIR3DS1 on the surface of NK cells. It also appears to affect the ligand specificity, licensing, or the ability of the NK cells to recognize virally infected cells, as evidenced by the differences in inhibition of HIV replication that are seen in individuals with different genotypes. Interestingly, KIR3DS1+ NK cells expand aggressively following acute infection in the presence of HLA-Bw4-80I, potentially allowing these anti-viral cytolytic effector cells to expand in sufficient numbers to gain effective control of the incoming virus [38]. However, based on the data presented here, individuals with increased numbers of effective copies of KIR3DL1 in the presence of KIR3DS1 may possess an enlarged pool of KIR3DS1+ NK cells prior to infection, which can potentially contribute to enhanced anti-viral control immediately upon transmission, without any proliferative delay to control HIV-1 replication, if their ligand is present. The fact that an increased KIR3DS1 effective count appears to impact relatively early measures of HIV-1 disease control, such as viral load at set point, reinforces the notion that elevated levels of KIR3DS1+ NK cells in acute infection may provide the needed effector cells to contain early viral replication until the HIV-specific CD8+ T cells are able to respond.

These observations add a new element to what is known about how genetic variation in the KIR locus modulates the immune response to HIV-1. It has already been shown that particular KIR variants interact with their ligands to influence control of HIV-1, with a strong interaction reported between some KIR3DL1 and HLA-B*5701 [13]. The novelty of our findings is that the counts of individual genes in the KIR locus directly influence early aspects of HIV-1 control, with individuals who have an effective copy of KIR3DS1, in combination with an effective copy of KIR3DL1, achieving the highest degree of viral suppression. This effect was first apparent from our association data and is strongly supported by functional experiments. In order to assess the possible implications of these findings for vaccine development, it is now a priority to elucidate the functional basis of how NK cells expressing sufficient quantities of both KIR3DL1 and KIR3DS1 suppress HIV-1, and in particular whether such suppression involves elements of adaptive immunity [46], or allow for the potential of specific recognition of infected cells by KIR3DS1+ NK cells that may drive viral evolution [12].

## Materials and Methods

### Ethics Statement

All samples used in this analysis were de-identified. Samples were received from collaborators at outside institutions and were approved under an IRB exemption by the Duke University Health System Institutional Review Board. The Massachusetts General Hospital institutional review board approved functional analyses, and each individual gave written informed consent for participation in the study.

### Participants

Participants were recruited from the Euro-CHAVI Consortium and from the Multicenter AIDS Cohort Study (MACS). All samples were consenting according to the IRB guidelines at their respective sites.

The Euro-CHAVI cohort represents a consortium of eight European and one Australian Cohorts/Studies that agreed to participate in the Host Genetic Core initiative of the Center for HIV-AIDS Vaccine Immunology (CHAVI). CHAVI is a consortium of universities and academic medical centers established by the National Institute of Allergy and Infectious Diseases, part of the Global HIV Vaccine Enterprise.

The Multicenter AIDS Cohort Study (MACS) is an ongoing prospective study of the natural and treated histories of HIV-1 infection in homosexual and bisexual men conducted by sites located in Baltimore, Chicago, Pittsburgh, and Los Angeles. A total of 6,973 men have been enrolled. From April 1984 through March 1985, 4,954 men were enrolled; an additional 668 men were enrolled from April 1987 through September 1991. A third enrollment of 1,351 men took place between October 2001 and August 2003. The 3,427 participants were HIV-seronegative at study entry and were tested for seroconversion semiannually by ELISA, with confirmation of positive tests by Western blotting.

A total of 978 healthy participants from the Boston area were typed for the KIR CNV. A total of 76 participants were recruited for this study.

### Set Point Determination

A seroconverting patient is defined as reaching set point after acute HIV infection, when at least two consecutive HIV-1 viral load values, taken at least a month apart, are within a 0.5 log range. All plasma viral load measurements within the set point range are then averaged to determine the phenotype [22]. A potential limitation is that our definition of set point may exclude some rapid progressors who never maintain a stable stage of infection.

### Structural Variants Determination

To call CNVs, we used PennCNV [23], a software that applies a hidden Markov-model-based approach for kilobase-resolution detection of CNVs from Illumina SNP genotyping data. PennCNV uses Log R ratio (LRR) and B allele frequency (BAF) measures automatically computed from the signal intensity files by BeadStudio, and we used the standard hg18 PennCNV hidden Markov model and population frequency of B allele (pfb) files. For data from the 1MDuo BeadChip, for which there is no standard pfb file, we used our own data to design a pfb file that would include the SNPs specific to this genotyping platform. For better handling of low-quality genotype data, we implemented GC-model signal pre-processing using standard files from PennCNV. We ran QC analysis on the samples and removed those with an LRR standard deviation greater than 0.28, a BAF median outside the range of 0.45 to 0.55, a BAF drift greater than 0.002, or a waviness factor not between −0.04 and 0.04 (*n* = 204). Samples were also removed if they were near threshold for more than one of the QC measurements, exhibited karyotype abnormalities, or were gross outliers for number of CNVs called (*n* = 59, total removals = 256 as some failed more than 1 QC parameter). CNV calls were restricted to autosomes. The CNV calls were prepared for regression analysis by creating separate duplication and deletion files, each containing a list of the SNPs that were deleted or duplicated, and indicating the number of copies the participant possessed (zero, one, two for deletion analysis; two, three, four for duplication analysis). The calls for each SNP were then run as genotypes in a regression using an additive genetic model, testing for association with HIV-1 set point. All samples that were determined to have a deletion or duplication of the *KIR3DS1-KIR3DL1* locus were visually inspected in BeadStudio to confirm the CNV.

### Determination of *KIR3DS1* and *KIR3DL1* Count

*KIR3DS1* and *KIR3DL1* copy number was measured using a quantitative real-time PCR assay. Primer sequences were: *KIR3DS1* forward primer, 5′- CTCGTTGGACAGATCCATGA -3′; *KIR3DS1* reverse primer, 5′- GTCCCTGCAAGGGCAC -3′; *KIR3DL1* forward primer, 5′- GCCTCGTTGGACAGATCCAT-3′; *KIR3DL1* reverse primer 5′- TAGGTCCCTGCAAGGGCAA-3′; *KIR3DL1-KIR3DS1* probe, 5′-VIC- GGGTCTCCAAGGCCAATTTCTCCAT-MGB-3′; *Beta-globin* forward primer, 5′- GGCAACCCTAAGGTGAAGGC -3′; *Beta-globin* reverse primer, 5′-GGTGAGCCAGGCCATCACTA-3′; *Beta-globin* probe, 5′-6FAM- CATGGCAAGAAAGTGCTCGGTGCCT-MGB-3′. Primers were purchased from Integrated DNA Technologies (Coralville, IA, USA), and probes were purchased from Applied Biosystems (Foster City, CA, USA). Since the sequences for *KIR3DL1* and *KIR3DS1* are so similar, we use the same probe for both *KIR3DL1* and *KIR3DS1*. Both reverse primers were validated in reference [47] as being specific for *KIR3DS1* and *KIR3DL1*, respectively. We designed new forward primers that create shorter products which are better suited to real-time PCR analysis. Concentration of DNA samples was determined by absorbance at 260 nm, and samples were diluted to achieve a concentration range of 1–20 ng/µL, of which 1 µL was used per reaction in a total volume of 10 µL per reaction.

Serially diluted DNA from the CEPH lines GM11840 and GM12752 was used as a standard, with concentrations ranging from ∼100 ng/µL to 8 pg/µL. Both lines have one *KIR3DL1* and one *KIR3DS1*, which was determined by running them against a standard that did not show copy number variability in the KIR region and that an external assay determined had both *KIR3DL1* and *KIR3DS1*. Thermal cycling was performed on the Applied Biosystems 7900 Sequence Detection System and data were captured using Sequence Detection System software v1.0. Cycling conditions were: 50°C×2 min, 95°C×10 min, followed by 40 cycles of two-step PCR with 15 s at 95°C and a 1 min extension at 60°C. The threshold ΔRn was set manually after visual inspection of the real-time PCR results. The cycle at which the threshold was achieved (Ct) for each CEPH standard reaction was plotted against the base 10 log of the input DNA amount, and the line of best fit through this standard curve was used to estimate the relative input amount for each gene (*KIR3DL1/KIR3DS1* or *Beta-globin*) in the unknown samples, which were run in duplicate. The lower limit of detection of the assay was approximately 16 pg of input DNA. Samples with estimated DNA input amounts of less than 16 pg, or for which the coefficient of variation (CV) in duplicate samples exceeded 0.25, were excluded from analysis. Samples with a CV greater than 0.1 were manually curated and were excluded if the results looked irregular for any reason (such as duplicates were not conclusive or a low DNA concentration). Thirty-nine samples (1.6%) were excluded due to low DNA concentration. The copy number of the unknown samples was estimated by the ratio of the *KIR3DL1* or *KIR3DS1* amount to the *Beta-globin* amount. Individual samples were then assigned a copy number by rounding the KIR/*Beta-globin* value to the nearest integer. Of the samples that had a high enough DNA concentration to use in the real-time assay, we were able to make 98.8% of the *KIR3DL1* calls and 98.4% of the *KIR3DS1* calls.

### *KIR3DL1* Allele Designations

The *KIR3DL1* alleles were characterized according to the protocol in ref. [47]. A total of 204 of our samples overlapped with the samples in the VL cohort in ref. [13].

We used the genealogy in ref. [21] to categorize all of the *KIR3DL1* alleles found in our sampled population. *KIR3DL1*-high: *001, *00101, *002, *015, *01501, *01502, *008, *009, *020, *022, *023, *029, *033, *035, *052; *KIR3DL1*-low: *005, *006, *007, *019, *028, *053, *054, 3DL1-Lv2, N9; KIR3DL1*004: *004, *00401, *00402.

*KIR3DL1*-surface includes all *KIR3DL1*-high and all *KIR3DL1*-low alleles.

Four samples were dropped due to an inconclusive allele type or because they had a rare *KIR3DL1* allele for which the quantity or presence of any cell surface expression was not known.

We were unable to count the number of *KIR3DL1*-high, *KIR3DL1*-low, and KIR3DL1*004 alleles for six samples where the *KIR3DL1* real-time assay counted three copies of *KIR3DL1*, since the allele genotyping assay is not quantitative and thus we could not discern if one of the alleles was duplicated. These samples were not included in the analysis.

### *HLA-B* Genotyping

*HLA-B* genotyping was performed by amplification of genomic DNA with primers that flank exons 2 and 3. PCR products are cleaned using Ampure (Beckman Coulter). The cleaned products are sequenced using appropriate nested primers. The sequenced products are cleaned using CleanSEQ (Beckman Coulter) and then run on the ABI PRIZM 3730. Sequence analysis is carried out using Assign (Conexio Genomics).

### Sample Selection

A total of 2,724 individuals with age and gender data were identified as whites by a principal component analysis of the genome-wide genotyping data (EIGENSTRAT method) and were eligible for the study. A total of 126 of these were dropped since they did not have a set point value. A total of 236 of these were dropped due to being outliers in the EIGENSTRAT analyses. This left 2,362 samples, of which 2,102 had Illumina genotype data and a sufficient PennCNV quality score to be included in the PennCNV analysis.

Of the 2,362 patients who had a set point value and were not EIGENSTRAT outliers, 1,751 had *KIR3DL1* and *KIR3DS1* real-time results. A total of 771 of these had *KIR3DL1* genotype data or had a *KIR3DL1* count of zero. Of these 771 patients, 48 could not be included because they were missing *HLA-B* data. Six samples were not included because the sum of *KIR3DL1+KIR3DS1* real-time counts did not equal the PennCNV call. Six samples were not included where the *KIR3DL1* real-time assay counted three copies of *KIR3DL1* (see previous section). Four samples were dropped where the *KIR3DL1* genotyping assay had two unique alleles and the real-time assay only counted one *KIR3DL1*. One sample was dropped for inconclusive *HLA-B* results.

Of the samples that were not EIGENSTRAT outliers, 738 samples had complete KIR3DS1 and KIR3DL1 effective calls, meaning that each had *KIR3DL1* and *KIR3DS1* real-time counts, *HLA-B* data, and *KIR3DL1* allele typing when the *KIR3DL1* assay showed the presence of at least one *KIR3DL1* allele. Of those with complete effective calls, 706 had stable HIV-1 viral load set point.

The individuals who were included in the NK cell inhibition assays were HIV-negative healthy controls from the Boston area. A total of 76 participants were recruited for these assays, including eight individuals expressing two copies of *KIR3DL1* and one copy of *KIR3DS1*. The numbers of included individuals with each genotype are listed in the legend for Figure 4.

### NK Cell Inhibition Assay

NK inhibition assays were performed as previously described [38]. Activated CD4^+^ T cells were generated from each donor for 4 d in culture with a bispecific antibody to CD3/CD8. The cells were then infected with a JRCSF (R5) at a multiplicity of infection of 0.01 for 4 h at 37°C. Cells were then washed twice, and 10^5^ CD4^+^ T cells were plated in quadruplicate in the presence of 50 U/ml IL-2. NK cells were enriched from whole blood by negative selection (RosetteSep, Stem Cell technologies) on the same day as CD4+ T cells were infected. NK cells were then added at a 10∶1 NK∶CD4 ratio. Supernatant was collected every 3–4 d for quantification of p24 Gag production by ELISA (p24 ELISA; Perkin Elmer). The percent inhibition was calculated as the difference between the level of p24 produced in wells containing medium alone and those also containing NK cells divided by the total level viral replication in medium alone wells.

### KIR3DS1/KIR3DL1+ NK Cell Frequencies and Transcriptional Levels

NK cell populations were isolated from peripheral blood mononuclear cells (PBMCs) by high-speed cell sorting using a fluorescence-activated cell sorter (BD FACSAria). For these cell-sorting experiments, PBMCs were purified from whole blood, which were then labeled with anti-CD3-phycoerythrin-Cy5.5 (anti-CD3-PE-Cy5.5), anti-CD56-PE-Cy7, anti-CD16-allophycocyanin-Cy7 (anti-CD16-APC-Cy7), anti-CD14-PE-Cy5, anti-CD19-PE-Cy5, DX9-FITC (KIR3DL1, BD Biosciences), and z27-PE (Beckman Coulter) antibodies. Gates were set to only include CD3^−^ CD14^−^ CD19^−^ CD56^+/−^ CD16^+/−^ NK cells, and all CD3^+^ CD14^+^ CD19^+^ cells were excluded. The average purity of sorted NK cell populations was 97.7% (range, 95.8% to 99.1%). Sorted NK cells were collected directly in RNA stabilizing buffer (RLT; Qiagen) and stored at −80°C. RNA was prepared using the RNeasy kit (Qiagen) and then used to prepare cDNA using the Superscript III kit (Invitrogen). All sorted events were recorded on the FACSAria and the frequency of CD3^−^ CD14^−^ CD19^−^ CD56^+/−^ CD16^+/^DX9^+^ (KIR3DL1+ NK cells) and CD3^−^ CD14^−^ CD19^−^ CD56^+/−^ CD16^+/^Z27^+^ DX9^−^ (KIR3DS1+ NK cells) were analyzed. The level of transcription of all KIRs was measured by quantitative PCR with SYBR green (Stratagene) as described previously [37]. To ensure specificity, dissociation curves were analyzed upon each run. The relative expression of KIR mRNA was normalized to the expression of glyceraldehyde-3-phosphate dehydrogenase (GAPDH) in sorted NK cell RNA preparations. The levels of KIR transcripts were then expressed as 2^50-cycle^ number above threshold.

### Statistical Analysis

The statistical analyses were performed using Stata/IC 10.0 for Windows. The association with set point was tested by using a linear regression of the effective gene count after correcting for age, gender, and ancestry by using the 12 significant EIGENSTRAT axes. Statistical significance refers to two-sided *p* values of<0.05.

### Accession Numbers

The NCBI (http://www.ncbi.nlm.nih.gov/sites/entrez) accession numbers for the sequences discussed in this article are: HLA-B (GeneID 3106; NC_000006.11), KIR3DL1 (GeneID 3811; NC_000019.9), KIR3DS1 (GeneID 3813).

## Supporting Information

Figure S1 *KIR3DL1* and *KIR3DS1* real-time quantification assays. Shown is a portion of the exon 4 sequences of all known alleles of KIR3DL1 (A) and KIR3DS1 (B). Alignment from the IPD-KIR database (http://www.ebi.ac.uk/ipd/kir/align.html). The assays for both genes used the same forward primer (red) and the same probe (orange). The T at position 543 is specific for *KIR3DL1* and *KIR3DS1*. The A at position 517 is specific to *KIR3DL1*, *KIR3DS1*, and *KIR3DL2*. *KIR3DL2* has multiple sequence differences in the space where the reverse primers (green) bind *KIR3DL1* and *KIR3DS1*. *KIR3DL1* and *KIR3DS1* diverge at position 566 in the reverse primer, where *KIR3DL1* has a T and *KIR3DS1* has a G. Images of amplification of *B-globin* (C), *KIR3DL1* (D), and *KIR3DS1* (E) real-time quantification assays.
(DOC)

Figure S2 High repeatability of real-time assay for *KIR3DL1* count and *KIR3DS1* count. A single plate of samples (*n* = 94) was run twice with the KIR real-time assays, as described in the Materials and Methods section. This figure shows a comparison of the replicates for *KIR3DL1* count (A) and *KIR3DS1* count (B).
(TIF)

Table S1 Duplications and deletions that associate with viral load set point.
(RTF)

Table S2 Raw gene counts from real-time assays.
(RTF)

Table S3 Concordance of real-time results with PennCNV results.
(RTF)

Table S4 Genotype frequencies of samples showing a duplication.
(RTF)

Table S5 *p* values for association with VL set point if patient is Bw6/Bw6.
(DOC)

Table S6 Frequency of specified *HLA-B* alleles among KIR-related groupings of *HLA-B* alleles.
(DOC)

Text S1 PennCNV versus real-time comparison.
(RTF)

## Abbreviations

CNV: copy number variant
HIV-1: human immunodeficiency virus type 1
KIR: killer cell immunoglobulin-like receptors
MHC: major histocompatibility complex
NK cells: natural killer cells
SNP: single-nucleotide polymorphism

## References

[1] Bashirova A. A, Martin M. P, McVicar D. W, Carrington M (2006). The killer immunoglobulin-like receptor gene cluster: tuning the genome for defense.. Annu Rev Genomics Hum Genet.

[2] Uhrberg M, Valiante N. M, Shum B. P, Shilling H. G, Lienert-Weidenbach K (1997). Human diversity in killer cell inhibitory receptor genes.. Immunity.

[3] Carrington M, Martin M. P, van Bergen J (2008). KIR-HLA intercourse in HIV disease.. Trends Microbiol.

[4] Martin M. P, Bashirova A, Traherne J, Trowsdale J, Carrington M (2003). Cutting Edge: expansion of the KIR locus by unequal crossing over.. J Immunol.

[5] Williams F, Maxwell L. D, Halfpenny I. A, Meenagh A, Sleator C (2003). Multiple copies of KIR 3DL/S1 and KIR 2DL4 genes identified in a number of individuals.. Hum Immunol.

[6] Qi Y, Martin M. P, Gao X, Jacobson L, Goedert J. J (2006). KIR/HLA pleiotropism: protection against both HIV and opportunistic infections.. PLoS Pathog.

[7] Martin M. P, Gao X, Lee J. H, Nelson G. W, Detels R (2002). Epistatic interaction between KIR3DS1 and HLA-B delays the progression to AIDS.. Nat Genet.

[8] Alter G, Martin M. P, Teigen N, Carr W. H, Suscovich T. J (2007). Differential natural killer cell-mediated inhibition of HIV-1 replication based on distinct KIR/HLA subtypes.. J Exp Med.

[9] Long B. R, Ndhlovu L. C, Oksenberg J. R, Lanier L. L, Hecht F. M (2008). Conferral of enhanced natural killer cell function by KIR3DS1 in early human immunodeficiency virus type 1 infection.. J Virol.

[10] Gaudieri S, DeSantis D, McKinnon E, Moore C, Nolan D (2005). Killer immunoglobulin-like receptors and HLA act both independently and synergistically to modify HIV disease progression.. Genes Immun.

[11] Barbour J. D, Sriram U, Caillier S. J, Levy J. A, Hecht F. M (2007). Synergy or independence? Deciphering the interaction of HLA Class I and NK cell KIR alleles in early HIV-1 disease progression.. PLoS Pathog.

[12] Alter G, Heckerman D, Schneidewind A, Fadda L, Kadie C. M (2011). HIV-1 adaptation to NK-cell-mediated immune pressure.. Nature.

[13] Martin M. P, Qi Y, Gao X, Yamada E, Martin J. N (2007). Innate partnership of HLA-B and KIR3DL1 subtypes against HIV-1.. Nat Genet.

[14] Kim S, Poursine-Laurent J, Truscott S. M, Lybarger L, Song Y. J (2005). Licensing of natural killer cells by host major histocompatibility complex class I molecules.. Nature.

[15] Fernandez N. C, Treiner E, Vance R. E, Jamieson A. M, Lemieux S (2005). A subset of natural killer cells achieves self-tolerance without expressing inhibitory receptors specific for self-MHC molecules.. Blood.

[16] Anfossi N, André P, Guia S, Falk C. S, Roetynck S (2006). Human NK cell education by inhibitory receptors for MHC class I.. Immunity.

[17] Altfeld M, Goulder P (2007). ‘Unleashed’ natural killers hinder HIV.. Nat Genet.

[18] Li H, Pascal V, Martin M. P, Carrington M, Anderson S. K (2008). Genetic control of variegated KIR gene expression: polymorphisms of the bi-directional KIR3DL1 promoter are associated with distinct frequencies of gene expression.. PLoS Genet.

[19] Pascal V, Yamada E, Martin M. P, Alter G, Altfeld M (2007). Detection of KIR3DS1 on the cell surface of peripheral blood NK cells facilitates identification of a novel null allele and assessment of KIR3DS1 expression during HIV-1 infection.. J Immunol.

[20] Gardiner C. M, Guethlein L. A, Shilling H. G, Pando M, Carr W. H (2001). Different NK cell surface phenotypes defined by the DX9 antibody are due to KIR3DL1 gene polymorphism.. J Immunol.

[21] Norman P. J, Abi-Rached L, Gendzekhadze K, Korbel D, Gleimer M (2007). Unusual selection on the KIR3DL1/S1 natural killer cell receptor in Africans.. Nat Genet.

[22] Fellay J, Shianna K. V, Ge D, Colombo S, Ledergerber B (2007). A whole-genome association study of major determinants for host control of HIV-1.. Science.

[23] Wang K, Li M, Hadley D, Liu R, Glessner J (2007). PennCNV: an integrated hidden Markov model designed for high-resolution copy number variation detection in whole-genome SNP genotyping data.. Genome Res.

[24] Price A. L, Patterson N. J, Plenge R. M, Weinblatt M. E, Shadick N. A (2006). Principal components analysis corrects for stratification in genome-wide association studies.. Nat Genet.

[25] Single R. M, Martin M. P, Meyer D, Gao X, Carrington M (2008). Methods for assessing gene content diversity of KIR with examples from a global set of populations.. Immunogenetics.

[26] Cella M, Longo A, Ferrara G. B, Strominger J. L, Colonna M (1994). NK3-specific natural killer cells are selectively inhibited by Bw4-positive HLA alleles with isoleucine 80.. J Exp Med.

[27] Gumperz J. E, Barber L. D, Valiante N. M, Percival L, Phillips J. H (1997). Conserved and variable residues within the Bw4 motif of HLA-B make separable contributions to recognition by the NKB1 killer cell-inhibitory receptor.. J Immunol.

[28] Gillespie G. M, Bashirova A, Dong T, McVicar D. W, Rowland-Jones S. L (2007). Lack of KIR3DS1 binding to MHC class I Bw4 tetramers in complex with CD8+ T cell epitopes.. AIDS Res Hum Retroviruses.

[29] Carr W. H, Rosen D. B, Arase H, Nixon D. F, Michaelsson J (2007). KIR3DS1, a gene implicated in resistance to progression to AIDS, encodes a DAP12-associated receptor expressed on NK cells that triggers NK cell activation.. J Immunol.

[30] O'Connor G. M, Guinan K. J, Cunningham R. T, Middleton D, Parham P (2007). Functional polymorphism of the KIR3DL1/S1 receptor on human NK cells.. J Immunol.

[31] Pando M. J, Gardiner C. M, Gleimer M, McQueen K. L, Parham P (2003). The protein made from a common allele of KIR3DL1 (3DL1*004) is poorly expressed at cell surfaces due to substitution at positions 86 in Ig domain 0 and 182 in Ig domain 1.. J Immunol.

[32] Kiepiela P, Leslie A. J, Honeyborne I, Ramduth D, Thobakgale C (2004). Dominant influence of HLA-B in mediating the potential co-evolution of HIV and HLA.. Nature.

[33] Gao X, Bashirova A, Iversen A. K, Phair J, Goedert J. J (2005). AIDS restriction HLA allotypes target distinct intervals of HIV-1 pathogenesis.. Nat Med.

[34] Carrington M, O'Brien S. J (2003). The influence of HLA genotype on AIDS.. Annu Rev Med.

[35] Gao X, Nelson G. W, Karacki P, Martin M. P, Phair J (2001). Effect of a single amino acid change in MHC class I molecules on the rate of progression to AIDS.. N Engl J Med.

[36] Hanke T, Takizawa H, Raulet D. H (2001). MHC-dependent shaping of the inhibitory Ly49 receptor repertoire on NK cells: evidence for a regulated sequential model.. Eur J Immunol.

[37] Held W, Dorfman J. R, Wu M. F, Raulet D. H (1996). Major histocompatibility complex class I-dependent skewing of the natural killer cell Ly49 receptor repertoire.. Eur J Immunol.

[38] Alter G, Rihn S, Walter K, Nolting A, Martin M (2009). HLA class I subtype-dependent expansion of KIR3DS1+ and KIR3DL1+ NK cells during acute human immunodeficiency virus type 1 infection.. J Virol.

[39] Pascal V, Yamada E, Martin M. P, Alter G, Altfeld M (2007). Detection of KIR3DS1 on the cell surface of peripheral blood NK cells facilitates identification of a novel null allele and assessment of KIR3DS1 expression during HIV-1 infection.. J Immunol.

[40] Fellay J, Ge D, Shianna K. V, Colombo S, Ledergerber B (2009). Common genetic variation and the control of HIV-1 in humans.. PLoS Genet.

[41] Cohen G. B, Gandhi R. T, Davis D. M, Mandelboim O, Chen B. K (1999). The selective downregulation of class I major histocompatibility complex proteins by HIV-1 protects HIV-infected cells from NK cells.. Immunity.

[42] Ward J, Davis Z, DeHart J, Zimmerman E, Bosque A (2009). HIV-1 Vpr triggers natural killer cell-mediated lysis of infected cells through activation of the ATR-mediated DNA damage response.. PLoS Pathog.

[43] Richard J, Sindhu S, Pham T. N, Belzile J. P, Cohen E. A (2010). HIV-1 Vpr up-regulates expression of ligands for the activating NKG2D receptor and promotes NK cell-mediated killing.. Blood.

[44] Shah A. H, Sowrirajan B, Davis Z. B, Ward J. P, Campbell E. M (2010). Degranulation of natural killer cells following interaction with HIV-1-infected cells is hindered by downmodulation of NTB-A by Vpu.. Cell Host Microbe.

[45] Kim S, Sunwoo J. B, Yang L, Choi T, Song Y. J (2008). HLA alleles determine differences in human natural killer cell responsiveness and potency.. Proc Natl Acad Sci U S A.

[46] Sun J. C, Beilke J. N, Lainer L. L (2009). Adaptive immune features of natural killer cells.. Nature.

[47] Martin M. P, Carrington M (2008). KIR locus polymorphisms: genotyping and disease association analysis.. Methods Mol Biol.

