# Correction: Copy Number Variation of KIR Genes Influences HIV-1 Control

**DOI:** 10.1371/annotation/7e17b146-a69c-4e83-9230-7340486d9dc8

**Published:** 2011-12-16

**Authors:** Kimberly Pelak, Anna C. Need, Jacques Fellay, Kevin V. Shianna, Sheng Feng, Thomas J. Urban, Dongliang Ge, Andrea De Luca, Javier Martinez-Picado, Steven M. Wolinsky, Jeremy J. Martinson, Beth D. Jamieson, Jay H. Bream, Maureen P. Martin, Persephone Borrow, Norman L. Letvin, Andrew J. McMichael, Barton F. Haynes, Amalio Telenti, Mary Carrington, David B. Goldstein, Galit Alter, on behalf of NIAID Center for HIV/AIDS Vaccine Immunology (CHAVI)

The affiliation for the twentieth author was incorrect. Mary Carrington is not affiliated with #10 but with #12 Cancer and Inflammation Program, Laboratory of Experimental Immunology, SAIC-Frederick, Inc., NCI-Frederick, Frederick, Maryland, United States of America. The author’s affiliation to #18 is unaffected.

