# Supplementary material for: Copy Number Variation of KIR Genes Influences HIV-1 Control
Source: PLoS Biol. 2011 Nov 29;9(11):e1001208. doi: 10.1371/journal.pbio.1001208 (PMC3226550; doi:10.1371/journal.pbio.1001208)

Figure S1A             510        520        530        540        550        560        570        580        590        600

***KIR3DL1* sequence** GCGCCTCGTT GGACAGATCC ATGATGGGGT CTCCAAGGCC AATTTCTCCA TCGGTCCCAT GATGCTTGCC CTTGCAGGGA CCTACAGATG CTACGGTTCT

3DL1*00101        A--------- ------A--- ---------- ---------- --T------- ---------- -----T---- ---------- ---------- ----------
 3DL1*00102        A--------- ------A--- ---------- ---------- --T------- ---------- -----T---- ---------- ---------- ----------
 3DL1*002          A--------- ------A--- ---------- ---------- --T------- ---------- -----T---- ---------- ---------- ----------
 3DL1*00401        A--------- ------A--- ---------- ---------- --T------- ---------- -----T---- ---------- ---------- ----------
 3DL1*00402        A--------- ------A--- ---------- ---------- --T------- ---------- -----T---- ---------- ---------- ----------
 3DL1*00501        A--------- ------A--- ---------- ---------- --T------- ---------- -----T---- ---------- ---------- ----------
 3DL1*00502        A--------- ------A--- ---------- ---------- --T------- ---------- -----T---- ---------- ---------- ----------
 3DL1*006          A--------- ------A--- ---------- ---------- --T------- -T-------- -----T---- ---------- ---------- ----------
 3DL1*007          A--------- ------A--- ---------- ---------- --T------- ---------- -----T---- ---------- ---------- ----------
 3DL1*008          A--------- ------A--- ---------- ---------- --T------- ---------- -----T---- ---------- ---------- ----------
 3DL1*009          A--------- ------A--- ---------- ---------- --T------- ---------- -----T---- ---------- ---------- ----------
 3DL1*01501        A--------- ------A--- ---------- ---------- --T------- ---------- -----T---- ---------- ---------- ----------
 3DL1*01502        A--------- ------A--- ---------- ---------- --T------- ---------- -----T---- ---------- ---------- ----------
 3DL1*016          A--------- ------A--- ---------- ---------- --T------- ---------- -----T---- ---------- ---------- ----------
 3DL1*01701        A--------- ------A--- ---------- ---------- --T------- ---------- -----T---- ---------- ---------- ----------
 3DL1*01702        A--------- ------A--- ---------- ---------- --T------- ---------- -----T---- ---------- ---------- ----------
 3DL1*018          A--------- ------A--- ---------- ---------- --T------- ---------- -----T---- ---------- ---------- ----------
 3DL1*019          A--------- ------A--- ---------- ---------- --T------- ---------- -----T---- ---------- ---------- ----------
 3DL1*020          AA-------- ------A--- ---------- ---------- --T------- ---------- -----T---- ---------- ---------- ----------
 3DL1*021          A--------- ------A--- ---------- ---------- --T------- ---------- -----T---- ---------- ---------- ----------
 3DL1*022          A--------- ------A--- ---------- ---------- --T------- ---------- -----T---- ---------- ---------- ----------
 3DL1*023          AA-------- ------A--- ---------- ---------- --T------- ---------- -----T---- ---------- ---------- ----------
 3DL1*024N         A--------- ----.-A--- ---------- ---------- --T------- ---------- -----T---- ---------- ---------- ----------
 3DL1*025          A--------- ------A--- ---------- ---------- --T------- ---------- -----T---- ---------- ---------- ----------
 3DL1*026          A--------- ------A--- ---------- ---------- --T------- ---------- -----T---- ---------- ---------- ----------
 3DL1*027          A--------- ------A--- ---------- ---------- --T------- ---------- -----T---- ---------- ---------- ----------
 3DL1*028          AA-------- ------A--- ---------- ---------- --T------- ---------- -----T---- ---------- ---------- ----------
 3DL1*029          A--------- ------A--- ---------- ---------- --T------- ---------- -----T---- ---------- ---------- ----------
 3DL1*030          A--------- ------A--- ---------- ---------- --T------- ---------- -----T---- ---------- ---------- ----------
 3DL1*031          A--------- ------A--- ---------- ---------- --T------- ---------- -----T---- ---------- ---------- ----------
 3DL1*032          A-------C- ------A--- ---------- ---------- --T------- ---------- -----T---- ---------- ---------- ----------
 3DL1*033          A--------- ------A--- ---------- ---------- --T------- ---------- -----T---- ---------- ---------- ----------
 3DL1*034          A--------- ------A--- ---------- ---------- --T------- ---------- -----T---- ---------- ---------- ----------
 3DL1*035          A--------- ------A--- ---------- ---------- --T------- ---------- -----T---- ---------- ---------- ----------
 3DL1*036          A--------- ------A--- ---------- ---------- --T------- ---------- -----T---- ---------- ---------- ----------
 3DL1*037          A--------- ------A--- ---------- ---------- --T------- ---------- -----T---- ---------- ---------- ----------
 3DL1*038          A--------- ------A--- ---------- ---------- --T------- ---------- -----T---- ---------- ---------- ----------
 3DL1*039          A--------- ------A--- ---------- ---------- --T------- ---------- -----T---- ---------- ---------- ----------
 3DL1*040          A--------- ------A--- ---------- ---------- --T------- ---------- -----T---- ---------- ---------- ----------
 3DL1*041          A--------- ------A--- ---------- ---------- --T------- ---------- -----T---- ---------- ---------- ----------
 3DL1*042          A--------- ------A--- ---------- ---------- --T------- ---------- -----T---- ---------- ---------- ----------
 3DL1*043          A--------- ------A--- ---------- ---------- --T------- ---------- -----T---- ---------- ---------- ----------
 3DL1*044          A--------- ------A--- ---------- ---------- --T------- ---------- -----T---- ---------- ---------- ----------

3DL1*054          A--------- ------A--- ---------- ---------- --T------- -----T---- -----G---- ---------- ---------- ----------
 3DL1*056          A--------- ------A--- ---------- ---------- --T------- ---------- -----T---- -------A-- ---------- ----------
 3DL1*057          AT-------- ------A--- ---------- ---------- --T------- ---------- -----T---- ---------- ---------- ----------

Figure S1B                510        520        530        540        550        560        570        580        590        600

***KIR3DS1* sequence** GCGCCTCGTT GGACAGATCC ATGATGGGGT CTCCAAGGCC AATTTCTCCA TCGGTTCCAT GATGCGTGCC CTTGCAGGGA CCTACAGATG CTACGGTTCT

3DS1*010          A--------- ------A--- ---------- ---------- --T------- -----T---- -----G---- ---------- ---------- ----------
 3DS1*011          A--------- ------A--- ---------- ---------- --T------- -----T---- -----G---- ---------- ---------- ----------
 3DS1*012          A-A------- ------A--- ---------- ---------- --T------- -----T---- -----G---- ---------- ---------- ----------
 3DS1*01301        A--------- ------A--- ---------- ---------- --T------- -----T---- -----G---- ---------- ---------- ----------
 3DS1*01302        A--------- ------A--- ---------- ---------- --T------- -----T---- -----G---- ---------- ---------- ----------
 3DS1*014          A--------- ------A--- ---------- ---------- --T------- -----T---- -----G---- ---------- ---------- ----------
 3DS1*045          A--------- ------A--- ---------- ---------- --T------- -----T---- -----G---- ---------- ---------- ----------
 3DS1*046          A--------- ------A--- ---------- ---------- -GT------- -----T---- -----G---- ---------- ---------- ----------
 3DS1*047          A--------- ------A--- ---------- ---------- --T------- --A--T---- -----G---- ---------- ---------- ----------
 3DS1*048          A--------- ------A--- ---------- ---------- --T------- -----T---- -----G---- ---------- ---------- ----------
 3DS1*049N         A--------- ------A--- ---------- ---------- --T------- -----T---- -----G---- ---------- ---------- ----------
 3DS1*055          A--------- ------A--- ---------- ---------- --T------- -----T---- -----G---- ---------- ---------- ----------

Figure S1: *KIR3DL1* and *KIR3DS1* real time quantification assays (begun on previous page, continued on next page)

Shown is a portion of the exon 4 sequences of all known alleles of KIR3DL1 (A) and KIR3DS1 (B). Alignment from the IPD-KIR database. (<http://www.ebi.ac.uk/ipd/kir/align.html>). The assays for both genes used the same forward primer (red) and the same probe (orange). The T at position 543 is specific for *KIR3DL1* and *KIR3DS1*. The A at position 517 is specific to *KIR3DL1, KIR3DS1* and *KIR3DL2. KIR3DL2* has multiple sequence differences in the space where the reverse primers (green) bind *KIR3DL1* and *KIR3DS1*. *KIR3DL1* and *KIR3DS1* diverge at position 566 in the reverse primer, where *KIR3DL1* has a T and *KIR3DS1* has a G. (Reverse primers validated in sup. ref. 2)

Highlighted in blue are places where an allele lists a sequence variation that overlaps with a primer or probe sequence:

3DL1*032 (2-4% frequency in Taiwanese (sup. ref. 3)

3DL1*056 (not listed in sup. ref. 3)

3DS1*046 (1-2% frequency in Hispanics (sup. ref. 3)

KIR3DL1*054 is a special case—a *KIR3DL1* allele, but where it shares the exon 4 sequence with *KIR3DS1*. This allele was recently discovered (sup. ref. 4) and only occurred once in ~6,000 samples that have been genotyped in that lab (personal communication from R. Thomas).

Images of amplification of *B-globin* (C), *KIR3DL1* (D), and *KIR3DS1* (E) real time quantification assays.


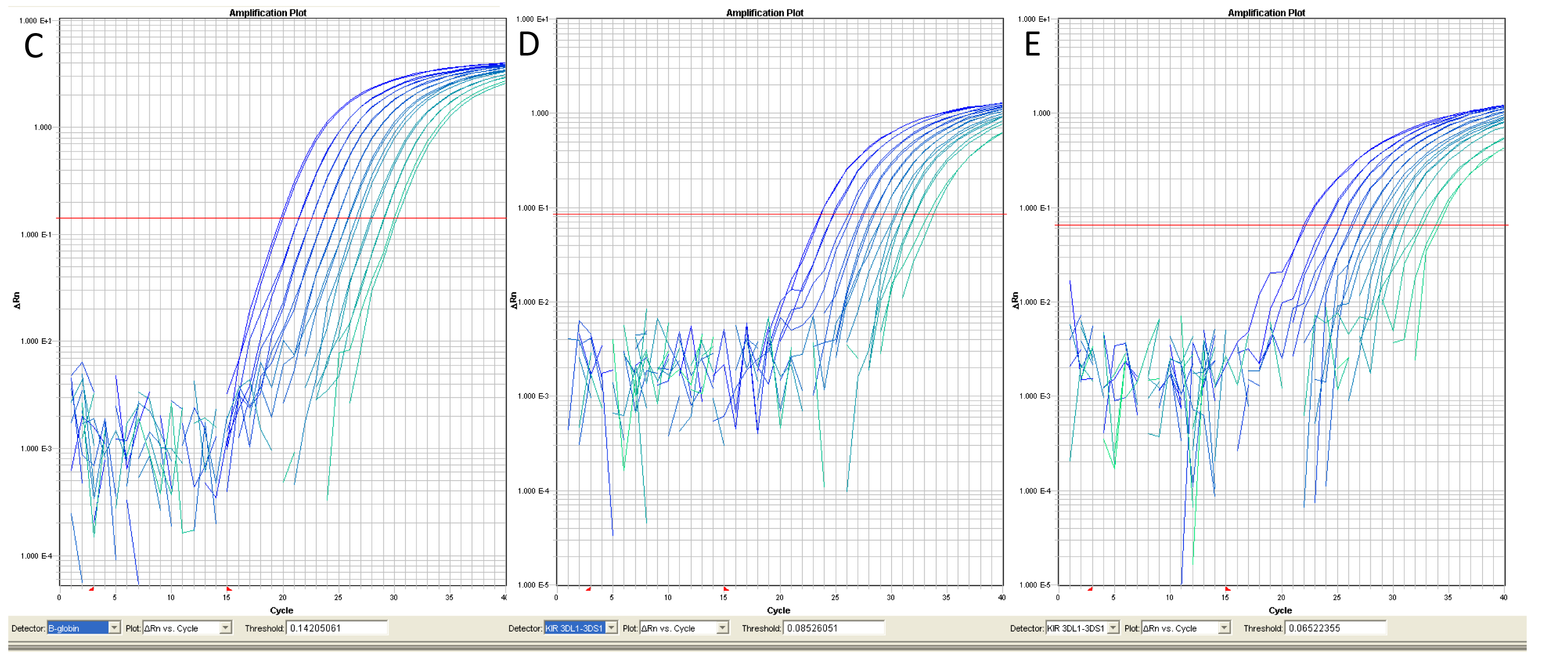

Supplement: Figure S1 — KIR3DL1 and KIR3DS1 real-time quantification assays. Shown is a portion of the exon 4 sequences of all known alleles of KIR3DL1 (A) and KIR3DS1 (B). Alignment from the IPD-KIR database (http://www.ebi.ac.uk/ipd/kir/align.html). The assays for both genes used the same forward primer (red) and the same probe (orange). The T at position 543 is specific for KIR3DL1 and KIR3DS1. The A at position 517 is specific to KIR3DL1, KIR3DS1, and KIR3DL2. KIR3DL2 has multiple sequence differences in the space where the reverse primers (green) bind KIR3DL1 and KIR3DS1. KIR3DL1 and KIR3DS1 diverge at position 566 in the reverse primer, where KIR3DL1 has a T and KIR3DS1 has a G. Images of amplification of B-globin (C), KIR3DL1 (D), and KIR3DS1 (E) real-time quantification assays. (DOC) [file pbio.1001208.s001.doc]
