# Supplementary figures and images for: Copy Number Variation of KIR Genes Influences HIV-1 Control
Source: PLoS Biol. 2011 Nov 29;9(11):e1001208. doi: 10.1371/journal.pbio.1001208 (PMC3226550; doi:10.1371/journal.pbio.1001208)

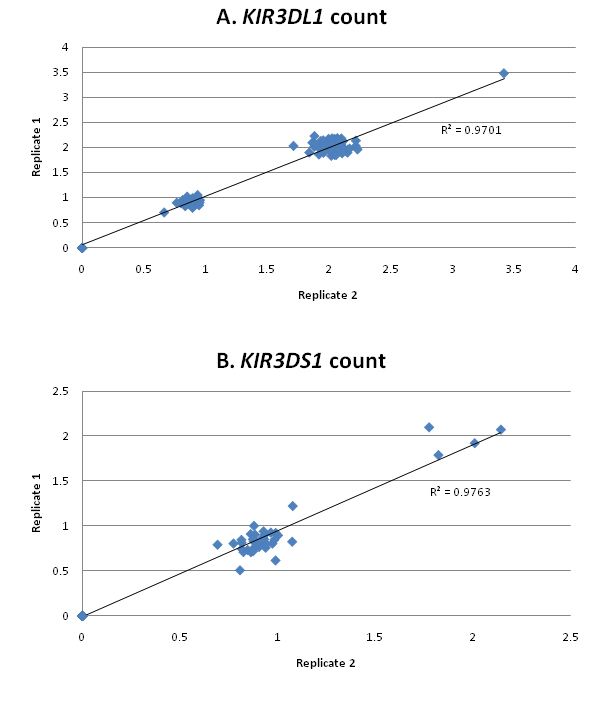

Supplement: Figure S2 — High repeatability of real-time assay for KIR3DL1 count and KIR3DS1 count. A single plate of samples (n = 94) was run twice with the KIR real-time assays, as described in the Materials and Methods section. This figure shows a comparison of the replicates for KIR3DL1 count (A) and KIR3DS1 count (B). (TIF) [file pbio.1001208.s002.tif]
