# Supplementary material for: Copy Number Variation of KIR Genes Influences HIV-1 Control
Source: PLoS Biol. 2011 Nov 29;9(11):e1001208. doi: 10.1371/journal.pbio.1001208 (PMC3226550; doi:10.1371/journal.pbio.1001208)
Supplement: Table S1 — Duplications and deletions that associate with viral load set point. (RTF) [file pbio.1001208.s003.rtf]

Table S1: Duplications and deletions that associate with viral load set point
This table includes all CNVs that occurred in more than 1% of our population, and that could be identified on all chip types.  The start and stop coordinates are approximate since there was often variability in the breakpoints of the CNVs, and because we are only able to resolve the breakpoint to the coordinate of the nearest SNP for which we had genotype data.  
Duplications							
Chr	Start	End	Beta of lowest p	MAF of lowest p	Lowest p	Overlapping/ included genes	
2	57260275	57299713	0.2323	0.01546	0.003992	-	
7	38308450	38333784	0.1988	0.02807	0.004096	TCRg	
19	60014787	60018551	-0.2401	0.01998	0.007303	KIR3DL1, KIR2DS2, KIR2DL4	
10	117218784	117231488	0.1973	0.01855	0.01278	ATRNL1	
3	8832963	8832963	0.3856	0.006898	0.01319	-	
7	76023564	76038186	0.385	0.004757	0.03938	-	
							
Deletions							
Chr	Start	End	Beta of lowest p	MAF of lowest p	Lowest p	Overlapping/ included genes	
6	31389749	31390117	-0.1825	0.06946	0.000212	HLA-B*	
3	89753046	89758560	0.3767	0.01284	0.000657	-	
7	38295538	38328597	-0.1747	0.04567	0.006287	TCRg	
11	55139733	55179162	-0.07757	0.1798	0.01121	OR4P4, OR4S2	
3	89752272	89758560	0.4421	0.004995	0.01528	-	
2	4191253	4200019	0.1272	0.04591	0.01786	-	
7	3577441	3584669	0.3792	0.005947	0.01887	SDK1	
15	75096474	75102029	-0.3564	0.007136	0.02016	PSTPIP1	
4	1.16E+08	116393377	-0.1714	0.03045	0.02302	-	
20	43796156	43811984	0.403	0.004995	0.02724	-	
12	69160993	69162296	0.1485	0.02022	0.02736	-	
19	60014787	60018551	0.2359	0.01427	0.02771	KIR3DL1, KIR2DS2, KIR2DL4	
4	92499956	92500442	0.2914	0.008801	0.03021	FAM190A	
14	85357100	85379917	0.2304	0.01451	0.03097	-	
5	28860345	28860345	-0.3818	0.005233	0.03225	-	
8	3774231	3777675	-0.1299	0.04329	0.03505	CSMD1	
8	7356405	7356405	0.2122	0.01665	0.03779	-	
12	1.3E+08	130378451	-0.2738	0.009753	0.03701	-	
16	2639305	2639305	0.1337	0.02355	0.04074	-	
* Driven by linkage disequilibrium between the CNV, rs9264942 and rs2395029 (sup. ref. 1)  
