# Supplementary material for: Copy Number Variation of KIR Genes Influences HIV-1 Control
Source: PLoS Biol. 2011 Nov 29;9(11):e1001208. doi: 10.1371/journal.pbio.1001208 (PMC3226550; doi:10.1371/journal.pbio.1001208)
Supplement: Table S2 — Raw gene counts from real-time assays. (RTF) [file pbio.1001208.s004.rtf]

Table S2: Raw gene counts from real time assays

		Raw KIR3DL1 count
	0	1	2	3	
0	2	53	997	13	
1	9	540	28	1	
2	63	34	2	0	
3	6	3	0	0	
