# Supplementary material for: Copy Number Variation of KIR Genes Influences HIV-1 Control
Source: PLoS Biol. 2011 Nov 29;9(11):e1001208. doi: 10.1371/journal.pbio.1001208 (PMC3226550; doi:10.1371/journal.pbio.1001208)
Supplement: Table S3 — Concordance of real-time results with PennCNV results. (RTF) [file pbio.1001208.s005.rtf]

Table S3: Concordance of real time results with PennCNV results

Real Time: KIR3DL1 count + KIR3DS1 count
	0	1	2	3	4	
0	2	0	0	0	0	
1	0	49	0	0	0	
2	0	4 *	1441	7 *	0	
3	0	0	0	60	0	
4	0	0	0	0	4	

* Samples where the real time data did not agree with the copy number state in PennCNV
