# Supplementary material for: Copy Number Variation of KIR Genes Influences HIV-1 Control
Source: PLoS Biol. 2011 Nov 29;9(11):e1001208. doi: 10.1371/journal.pbio.1001208 (PMC3226550; doi:10.1371/journal.pbio.1001208)
Supplement: Table S4 — Genotype frequencies of samples showing a duplication. (RTF) [file pbio.1001208.s006.rtf]

Table S4: Genotype frequencies of samples showing a duplication
KIR3DL1 count	KIR3DS1 count	Frequency
(% of total samples with a duplication)	
0	3	6.0	
1	2	38.5	
1	3	3.6	
2	1	33.7	
2	2	2.4	
3	0	14.5	
3	1	1.2	
83 samples had a duplication, KIR3DL1 and KIR3DS1 real time counts, and were not EIGENSTRAT outliers. 
