# Supplementary material for: Copy Number Variation of KIR Genes Influences HIV-1 Control
Source: PLoS Biol. 2011 Nov 29;9(11):e1001208. doi: 10.1371/journal.pbio.1001208 (PMC3226550; doi:10.1371/journal.pbio.1001208)
Supplement: Table S5 — p values for association with VL set point if patient is Bw6/Bw6. (DOC) [file pbio.1001208.s007.doc]

Table S5: p-values for association with VL set point if patient is Bw6/Bw6

|  | n | p  Alone ^a^ | n | p  In combined model |
| --- | --- | --- | --- | --- |
| *KIR3DS1* count | 239 | 0.662 | 106 | 0.321 ^b^ |
| *KIR3DL1-surface* count | 106 | 0.424 | 106 | 0.214 ^b^ |

^a^ Model includes age, gender, 12 EIGENSTRAT axes and one KIR gene.

^b^ Model includes age, gender, 12 EIGENSTRAT axes, *KIR3DS1* count, *KIR3DL1-surface* count.
