# Supplementary material for: Copy Number Variation of KIR Genes Influences HIV-1 Control
Source: PLoS Biol. 2011 Nov 29;9(11):e1001208. doi: 10.1371/journal.pbio.1001208 (PMC3226550; doi:10.1371/journal.pbio.1001208)
Supplement: Table S6 — Frequency of specified HLA-B alleles among KIR-related groupings of HLA-B alleles. (DOC) [file pbio.1001208.s008.doc]

Table S6: Frequency of specified *HLA-B* alleles among KIR-related groupings of *HLA-B* alleles

|  | Bw4-80I present | Bw4-80T present | Bw6/Bw6 |
| --- | --- | --- | --- |
| HLA-B*57 | 26.3% | 6.8% | 0% |
| HLA-B*27 | 5.5% | 17.1% | 0% |
| HLA-B*35Px | 6.2% | 5.0% | 14.9% |

This table includes the patients in the set point analysis with complete KIR3DL1 and KIR3DS1 effective calls.
