# Supplementary material for: Copy Number Variation of KIR Genes Influences HIV-1 Control
Source: PLoS Biol. 2011 Nov 29;9(11):e1001208. doi: 10.1371/journal.pbio.1001208 (PMC3226550; doi:10.1371/journal.pbio.1001208)
Supplement: Text S1 — PennCNV versus real-time comparison. (RTF) [file pbio.1001208.s009.rtf]

Supplementary Results

PennCNV vs Real Time Comparison: 
Since the PennCNV results showed multiple CNVs in the KIR region, not all of the CNV calls would be expected to agree with the sum of the real time counts specific for KIR3DL1 + KIR3DS1.  However, there are 3 SNPs (rs631717, rs649216, and rs581623) that fall in or near this locus which can be used to check the real time results.  Of the samples that had both a PennCNV result and a real time result for both genes, the copy number state at these 3 SNPs agreed with the sum of KIR3DL1+KIR3DS1 from the real time assay in 98.7% of the samples (1546/1567).  We inspected the BeadStudio files for all of the samples where the copy number state at these 3 SNPs did not agree with the sum of KIR3DL1+KIR3DS1 from the real time assay, and found that the real time results were accurate for 10 of the samples.  There were 11 samples (0.7%) where the results still did not agree after inspection (Table S3, starred), and these discrepancies were dropped from the analysis.  


References for Supplementary Materials:
1	Fellay J, Shianna KV, Ge D, Colombo S, Ledergerber B, et al. (2007) A whole-genome association study of major determinants for host control of HIV-1. Science  317: 944-947. 
2	Martin MP, Carrington M (2008) KIR locus polymorphisms: genotyping and disease association analysis.  Methods Mol Biol 415: 49-64.  
3	Norman PJ, Abi-Rached L, Gendzekhadze K, Korbel D, Gleimer M, et al. (2007) Unusual selection on the KIR3DL1/S1 natural killer cell receptor in Africans.  Nat Genet  39: 1092-1099.  
4	Thomas R, Yamada E, Alter G, Martin MP, Bashirova AA, et al. (2008) Novel KIR3DL1 Alleles and Their Expression Levels on NK Cells: Convergent Evolution of KIR3DL1 Phenotype Variation?  J Immunol 180: 6743-6750.
